# The dose effectiveness of extracorporeal shockwave on plantar flexor spasticity of ankle in stroke patients: a randomized controlled trial

**DOI:** 10.1186/s12984-024-01473-z

**Published:** 2024-10-01

**Authors:** Shu-Mei Yang, Yen-Hua Chen, You-Lin Lu, Chueh-Hung Wu, Wen-Shiang Chen, Meng-Ting Lin

**Affiliations:** 1 Department of Physical Medicine and Rehabilitation, National Taiwan University Hospital Hsin-Chu Branch, Hsinchu, Taiwan; 2 Department of Physical Medicine and Rehabilitation, National Taiwan University Hospital, College of Medicine, National Taiwan University, Taipei, Taiwan

**Keywords:** Extracorporeal shockwave therapy, Stroke, Spasticity

## Abstract

**Background:**

Extracorporeal shockwave therapy (ESWT) has been proven beneficial for post-stroke spasticity (PSS) of ankle plantar flexor muscles. This study aims to investigate the dose-response effectiveness of focused-ESWT and the duration of its effect on the treatment of ankle PSS in stroke patients.

**Methods:**

In this double-blinded randomized controlled trial, stroke patients diagnosed with PSS in the ankle plantar flexor muscles were randomly assigned to two groups. The experimental group received double-dose ESWT (4000 pulses per session) targeting spastic calf muscles, while the control group received half the dose (2000 pulses per session). Both groups underwent four sessions over two weeks. The outcomes, including modified Ashworth Scale (MAS), modified Tardieu Scale (MTS), passive range of motion (PROM) of the ankle, Timed Up and Go (TUG) Test, Barthel index and strain elastography were evaluated at baseline, 1st, 4th, 12th, and 24th week after ESWT.

**Results:**

Within-group analysis revealed significant improvements in MAS, PROM, TUG Test, and Barthel index for the double-dose ESWT group and improvements in Barthel index for the control group. Between-group analysis revealed greater improvements in TUG Test, Barthel Index and strain elastography for the double-dose ESWT group. Generalized estimating equations analysis indicated that the double-dose ESWT group achieved superior outcomes in the TUG Test, Barthel Index, and strain elastography across various time points and groups.

**Conclusions:**

Double-dose ESWT showed better functional improvement and elastography compared to the control group. ESWT demonstrated dose-response effectiveness for PSS of ankle-equinus.

**Trial registration:**

NCT05878223.

## Introduction

Spasticity is a common post-stroke complication that arises from upper motor neuron syndrome and manifests as velocity-dependent elevated muscle tone due to heightened muscle spindle excitability [1]. This condition appears in varying degrees anytime from days to 18 months post-stroke [2, 3]. The incidence of post-stroke spasticity (PSS) varies widely, with reports indicating it affects between 4 and 50% of people within 6 months of experiencing a stroke [4]. PSS significantly impedes neurological recovery, daily self-care, and quality of life, imposing substantial burdens on those affected and their caregivers [5]. 

Traditionally, PSS management encompassed a range of conservative treatments, including oral medication, physical therapy, orthotic devices, and local botulinum toxin injections [6]. Notably, extracorporeal shockwave therapy (ESWT) has recently emerged as a viable treatment modality [7]. 

Characterized by its high-energy mechanical waves, ESWT stimulates injured tissues to promote neovascularization, enhance blood circulation, facilitate cellular self-repair mechanisms, and interrupt pain signal transmission for an analgesic effect [8]. ESWT has been used to treat chronic musculoskeletal diseases such as calcific rotator cuff tendinitis, lateral epicondylitis, and plantar fasciitis. It may reduce disease recurrence, alleviate pain, and enhance functionality [9]. The mechanisms behind ESWT’s reduction of spasticity may involve several physiological effects [10]. Shock wave pressure can break the functional link between actin and myosin, reducing muscle stiffness and allowing forces to be transmitted to muscle spindles, which decreases spinal cord excitability [11]. Additionally, ESWT induces nitric oxide production, enhancing neovascularization and improving muscle stiffness [12]. ESWT also reduces motor neuron excitability and the number of acetylcholine receptors at neuromuscular junctions, leading to temporary dysfunction of nerve conduction [13]. Studies have shown that ESWT improves the rheological properties and trophic conditions of spastic muscles, contributing to reduced spasticity [14]. Clinical studies in people who have had a stroke validated the effectiveness of ESWT in reducing spasticity and indicated it has comparable effectiveness to botulinum toxin injections in managing PSS [15–17]. 

Post-stroke, many survivors face excessive spasticity of the ankle plantar flexors, leading to compromised dorsiflexor muscle strength, poor ankle joint control, abnormal gait patterns, increased energy expenditure during walking, localized ankle pain, and an elevated fall risk [18]. Previous studies have predominantly focused on the gastrocnemius and/or soleus muscles, demonstrating improved Modified Ashworth Scale (MAS) and Timed Up and Go (TUG) test scores, reduced pain, and enhanced passive ankle range of motion [19–22]. 

Previous research has not yet established the optimal treatment protocols for ESWT in stroke rehabilitation [23]. Specifically, there is a lack of evidence regarding the most effective type of ESWT (focused or radial), treatment parameters (intensity, frequency, and number of sessions), and the duration of ESWT’s effects on spasticity. Research has shown that ESWT can reduce spasticity for at least four to six weeks in patients with stroke or cerebral palsy [24, 25]. Another study on the long-term effects of ESWT indicated that reductions in pain and MAS grades, as well as improvements in motor function, persisted for up to 12 weeks [26]. 

There are two main types of ESWT: focused ESWT and radial ESWT. Focused ESWT is generated by electromagnetic, electrohydraulic, and piezoelectric sources, allowing it to penetrate tissues as deep as 12 cm with minimal damage to the skin and underlying soft tissues. In contrast, radial ESWT, generated by a pneumatic system, penetrates tissues only 3–4 cm deep. Overall, focused ESWT delivers higher intensity within a specific target area and deep penetration capabilities, whereas radial ESWT affects a broader but more superficial region [27]. Despite these distinctions, there is no definitive evidence indicating which type of ESWT is more effective in treating spasticity [27]. Most previous studies have investigated radial ESWT, while the effects of focused ESWT remain less explored [20, 21, 28]. Prior research suggests that both focused and radial ESWT can yield significant reductions in spasticity, improvements in ankle passive range of motion (PROM), and dynamic plantar contact area on the affected foot in stroke patients with spastic equinus foot [29]. However, no significant difference was observed in changes in either MAS scores or Tardieu angles between the two groups. The research also indicates that radial shock wave therapy seems to yield greater improvement in ankle PROM and dynamic plantar contact area on the affected foot [29]. Although prior research has examined the effects of focused versus radial ESWT on spastic equinus in stroke patients and identified effective application sites such as the myotendinous junction or the muscle belly [20, 29], there was no study comparing the impact of varying doses of ESWT on ankle plantar flexor spasticity in this population.

Therefore, the present study aims to explore the dose-response effectiveness of focused ESWT on post-stroke ankle plantar flexor spasticity and investigate the duration of its effects. This study employs a randomized controlled trial design to determine the optimal dosage for maximizing therapeutic outcomes in stroke survivors. We hypothesized that doubling the shockwave dosage for treating PSS would result in greater reduction of spasticity, improved function, and decreased muscle stiffness as evaluated by elastography; the effect of double-dose shockwave would last for up to 6 months.

## Methods

### Study design

This was a prospectively registered, double-blinded, randomized controlled trial with concealed allocation, blinded assessors and intention-to-treat analysis. The trial was conducted from January 2022 to April 2024 in a tertiary-referral medical center in Taiwan. The research was approved by the hospital’s research ethics committee and adhered to the principles of the Declaration of Helsinki. All participants provided informed consent.

The physiotherapist administered the shockwave therapy and the independent study coordinator was responsible for participant randomization and allocation; both were aware of the treatment groups. The other independent physiotherapist performed the outcome assessment, blinded to the randomization and the treatment procedure. Randomization was conducted using permuted blocks of four from a computer-generated random sequence, and the allocation results were sealed in masked envelopes. Each consecutive envelope was opened at the time of enrollment. Participants were then allocated to either the double-dose ESWT group or the control ESWT group to receive sequential ESWT treatment.

### Patients

The inclusion criteria were: individuals with (1) unilateral hemisphere cerebral stroke aged 20 years or older; (2) ankle plantar-flexor muscle spasticity greater than grade one, evaluated via the MAS; and (3) stable vital signs and clinical condition. Exclusion criteria were: individuals with (1) recurrent cerebral stroke, traumatic brain injury, brain tumor, or other brain-related diseases; (2) other central nervous system diseases (e.g., spinal cord injury or Parkinson’s disease) or musculoskeletal disorders that could impact muscle spasticity assessments; (3) malignant tumors, coagulation disorders, infections, or pacemakers; and (4) impaired cognition or aphasia. We additionally excluded patients who (5) have undergone ESWT or received botulinum toxin injections for plantar flexor spasticity in the past three months.

### Interventions

In this study, eligible patients were allocated to the double-dose shockwave group or the control shockwave group. We used a focused ESWT device PiezoWave2 (Richard Wolf GmbH, Knittlingen, Germany). One physiotherapist with 10 years of experience administered the ESWT to all participants, who was not involved in baseline evaluation or any follow-up assessment. The double-dose ESWT group received focused ESWT to the gastrocnemius and soleus muscles on the spastic side (2000 shots for each muscle, totaling 4000 shots per session, targeting prominent motor end-plates where the gastrocnemius muscle was located at the proximal one-third of the leg and the soleus at approximately the middle of the leg). The control ESWT group received focused ESWT to the spastic gastrocnemius muscles (a total of 2000 shots per session). Both groups underwent a total of four ESWT sessions, twice a week for two consecutive weeks.

The focused ESWT was set to a frequency of 4 Hz and an energy flux density (i.e., intensity) of 0.10-0.134 mJ/mm^2^. The ESWT was applied to the gastrocnemius and soleus muscles at depths determined by B-mode ultrasound (SONIMAGE HS2, Konica Minolta, Tokyo, Japan) for precision target of motor points in the spastic calf muscles. The focusing pads with eight different depths ranging from 5 to 40 mm were chosen accordingly. Gel was used on the skin-pad and pad-probe interfaces to enhance energy transmission. No local anesthesia was applied during ESWT. After the injection, all participants were allowed to take paracetamol, but not non-steroidal anti-inflammatory medications. All patients received traditional rehabilitation, which involves range of motion exercises, muscle stretching and strengthening, stance and balance training, core stability exercises, gait training, functional training, the use of physical modalities, and orthoses [30, 31]. 

### Outcome measures

The outcome measurements were performed by a highly experienced physiotherapist with 10 years of experience and was not involved in applying the shockwave therapy. This physiotherapist was blinded to the treatment allocation. The measurements were taken at baseline, week 1, week 4, week 12, and week 24. The primary outcome was the ankle plantar flexor muscle’s MAS score. The MAS was utilized to semi-quantify resistance during muscle stretch. It features six grades, ranging from 0 to 4 (including 1+). A higher MAS indicates heightened muscle tone [32]. Participants assumed a prone position with a fully extended knee and maintained their ankle in a neutral position. The participants extended their ankles from the potential maximal plantarflexion position to the maximal dorsiflexion position.

The secondary outcome measures included the Modified Tardieu Scale (MTS) angles, ankle ROM, the TUG test, the Barthel index, and strain elastography of the plantar flexor muscles. The MTS angles included R2 and R1, signifying the angle of the slow passive stretch and the catch angle of the fast passive stretch, respectively. The discrepancies between R2 and R1 were indicative of muscle spasticity [33]. MTS assessments were conducted with the participants positioned similar to MAS. Ankle PROM was measured via goniometry, where the neutral ankle position was considered to be zero degrees. Ankle dorsiflexion was recorded as a positive degree, while plantarflexion was a negative degree. The TUG test was employed to assess movement and balance capabilities during standing up and walking [34]. The test began with the participant in a seated position, after which they were instructed to stand up, walk for 3 m, turn around, and return to a seated position upon the therapist’s command. The time from the moment the patient started standing until they were seated again was recorded [34]. The Barthel Index is an ordinal scale used to assess functional independence ranging from 0 to 100, with scoring intervals of 5. It encompasses 10 skills related to activities of daily living (ADL) [35]. 

Strain elastography of the plantar flexor muscles was conducted using the B-mode and elastography mode ultrasonography with the “L18-4” linear probe (SONIMAGE HS2, Konica Minolta, Tokyo, Japan), administered by an independent physiatrist with five years of sonographic and relevant elastographic training. Intra-rater reliability was assessed before the trial began. Patients were instructed to maintain a prone position, consistent with the MAS and MTS settings. To minimize variability, all measurements were taken at the same position on the spastic medial gastrocnemius muscle. The muscle under examination was initially scanned using B-mode ultrasound in the transverse view to confirm the position, after which the elastography mode was employed for further assessment. The examiner applied compressional force to the probe, alternating with relaxing it at regular intervals. The elastographic images were generated with a consistent color presentation of tested tissues and stable strain graph during the rhythmical compression-relaxation cycles.

Color images transitioned from red (indicating hardness) to blue (indicating softness), representing the tested tissue’s relative strain. Strain elastography quantifies the strain ratio between treated muscles and a reference object using the formula Strain Ratio (SR) = 𝜀_muscle_ / 𝜀_reference_, where a higher SR indicates a more resistant muscle [36]. The Aquaflex gel pads from Parker Laboratories, Fairfield, NJ, USA, served as the reference object. The region of interest (ROI) was defined as 4 mm × 30 mm for the reference object and 18 mm × 30 mm for the medial gastrocnemius muscle [36]. ROI was measured three times to calculate the average SR of the examined medial gastrocnemius muscle.

### Data analysis

The sample size was determined using G-power 3.1.9.4 (University of California, Los Angeles) and preliminary power analysis. To achieve sufficient power with an effect size of 0.41, a power of 0.8, α of 0.05, and a loss rate of 10%, data from at least 16 participants in each group were required. The mean and standard deviation (SD) were presented for continuous data; the medians and interquartile ranges (IQRs) were used for ordinal variables and the percentages for categorical variables. The Shapiro-Wilks test assessed normal distribution, and the Mann-Whitney U test compared non-parametric data between groups. The Friedman test was employed for the repeated measurements of non-parametric comparisons. Generalized Estimation Equation (GEE) analysis was conducted for between-time, between-group, and group-time interaction. All statistical tests were two-tailed, with a *p* < 0.05 considered statistically significant. IBM SPSS Statistics Version 22 was used for all data analyses.

## Results

### Flow of participants through the study

**Fig. 1:**
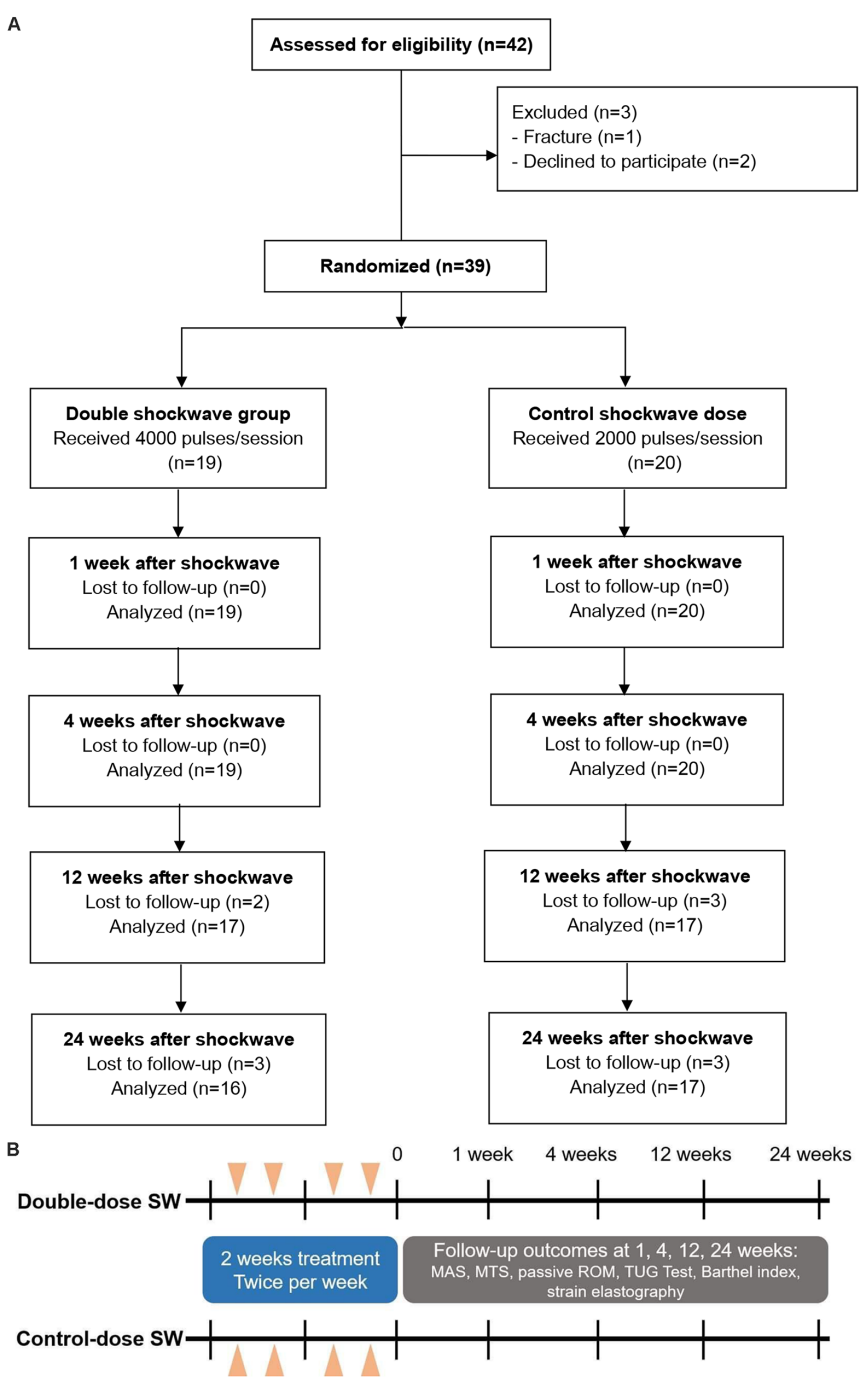
(**A**) Flow of participants through the trial; (**B**) Timeline of intervention and follow-up process

A total of 42 participants were initially assessed for eligibility, and three were excluded (Fig. 1). Consequently, 39 participants with PSS at the ankle plantar flexor muscle were included and randomized into two groups: the experimental (double-ESWT dose) group, consisting of 19 participants, and the control ESWT group, consisting of 20 participants. No significant adverse effects were reported throughout the study. The timeline of intervention and follow-up process were described in Fig. 1B.

### Baseline characteristics

The baseline demographic and clinical characteristics of the study participants are presented in Table 1. Among the 39 patients recruited, the youngest was 33 years old and the oldest was 84 years old. The mean age of patients in the double-dose ESWT group was 60.3 (SD: 14.3) years, while the mean age of patients in the single-dose ESWT group was 64.4 (SD: 10.3) years. No significant differences were found in age, gender, stroke duration, pre-existing conditions, stroke type, affected limbs, MAS scores, MTS, PROM, TUG test, or the Barthel index between the two groups.

**Table 1:** Baseline characteristics of the study participants

Characteristic		Double SW group (*n* = 19)	Control SW group (*n* = 20)	*p*-value^†^
Age (years), mean (SD)		60.3 (14.3)	64.4 (10.3)	0.359
Female (%)		31.3	38.9	0.407
Hypertension (%)		87.5	88.9	0.926
DM (%)		18.8	27.8	0.558
Stroke onset (months), mean (SD)		17.3 (36.5)	22.8 (23.4)	0.595
Ischemic stroke (%)		68.8	38.9	0.089
Affected limb at left side (%)		43.8	66.7	0.193
MAS, median (IQR)		2.63 (1.09)	2.56 (0.92)	0.815
R1 angle of MTS (degrees), mean (SD)		29.69 (8.26)	31.11(10.79)	0.793
R2 angle of MTS (degrees), mean (SD)		46.13 (8.73)	43.61(9.52)	0.411
R2-R1 angle of MTS (degrees), mean (SD)		16.44 (8.82)	12.50 (11.01)	0.262
PROM (degrees), mean (SD)		44.67 (9.57)	44.72 (9.31)	0.986
Timed Up and Go Test (seconds), mean (SD)		44.24 (38.82)	39.04 (29.71)	0.621
Barthel index, mean (SD)		68.13 (26.20)	66.39 (22.67)	0.742
Strain elastography, mean (SD)		1.09 (0.34)	1.10 (0.50)	0.846

† Between-group comparison: Mann-Whitney U test was used for statistical analysis

Abbreviation: DM, diabetes mellitus; MAS, Modified Ashworth Scale; MTS, Modified Tardieu Scale; PROM, passive range of motion; R1, angle of catch seen at quick speed; R2, Full range of motion at slow release of muscle; SD, standard deviation; SW, shockwave

### Outcomes

The effectiveness of ESWT on ankle plantar flexor spasticity post-stroke over 24 weeks is demonstrated in Table 2. For the primary outcome MAS, significant within-group improvements were observed in the double ESWT group over 24 weeks (*p* = 0.043), while the control ESWT group showed no significant change (*p* = 0.128). As for the secondary outcomes, the double ESWT group exhibited significant PROM improvement from baseline to the 24-week follow-up, with a mean increase of 6.71 degrees (*p* = 0.007). No significant change was found in the control ESWT group for PROM (*p* = 0.181). The TUG test also showed significant improvement in the double ESWT group (*p* < 0.001), but was not seen in the control ESWT group (*p* = 0.114). For the Barthel index, a statistically significant improvement was observed in both groups. No significant differences were found between the groups in MTS or strain elastography.

**Table 2:** Improvement of outcomes in both group

Outcome	Baseline	Week 1	Week 4	Week 12	Week 24	*p*-value^†^
**Primary outcome: MAS**						
**Double SW group**	2.63 (1.09)	2.38 (0.89)	2.13 (1.02)	2.50 (1.03)	2.50 (1.03)	0.043*
**Control SW group**	2.56 (0.92)	2.22 (0.94)	2.22 (0.94)	2.44 (1.04)	2.39 (1.04)	0.128
**Secondary outcomes**						
**R2-R1 angle of MTS**						
**Double SW group**	16.44 (8.82)	18.31 (9.03)	15.94 (5.84)	19.19 (6.16)	17.50 (5.16)	0.285
**Control SW group**	12.50 (11.01)	15.83 (10.04)	13.06 (9.87)	15.56 (8.89)	16.94 (8.77)	0.338
**PROM**						
**Double SW group**	44.67 (9.57)	48.13 (9.11)	50.94 (10.04)	48.56 (11.54)	51.38 (8.54)	0.007**
**Control SW group**	44.72 (9.31)	49.17 (10.04)	47.78 (11.79)	48.06 (13.41)	49.72 (12.06)	0.181
**Timed Up and Go Test**						
**Double SW group**	44.24 (38.82)	34.21 (37.32)	40.16 (52.01)	31.95 (38.20)	30.95 (38.02)	<0.001**
**Control SW group**	39.04 (29.71)	37.61 (28.26)	37.80 (31.23)	34.42 (26.80)	35.88 (27.39)	0.114
**Barthel index**						
**Double SW group**	68.13 (26.20)	76.56 (18.14)	81.88 (16.42)	83.13 (15.15)	83.75 (15.22)	<0.001**
**Control SW group**	66.39 (22.67)	69.72 (22.33)	69.72 (22.33)	70.00 (22.43)	71.11 (23.42)	0.009**
**Strain elastography**						
**Double SW group**	1.09 (0.34)	0.94 (0.35)	1.01 (0.38)	1.07 (0.49)	0.98 (0.56)	0.385
**Control SW group**	1.10 (0.50)	1.41 (0.71)	0.99 (0.38)	1.04 (0.50)	1.00 (0.39)	0.231

† Friedman test analysis was used for repeated measurements of non-parametric comparison

The data was presented as mean (SD)

Abbreviation: MAS, Modified Ashworth Scale; SW: shockwave; MTS, Modified Tardieu Scale; PROM, passive range of motion; R1, angle of catch seen at quick speed; R2, Full range of motion at slow release of muscle; SD, standard deviation

*p < 0.05, **p < 0.01

In Table 3, we compared the mean changes from baseline between the double-dose ESWT group and the control ESWT group at four follow-up time points for the primary and secondary outcomes. For the primary outcome MAS and the secondary outcomes MTS and PROM, no significant difference between groups was noted in change from baseline to any follow-up time point. For the secondary outcome TUG test, there were between-group differences in which the double ESWT group showed greater, statistically significant improvements at all follow-up points, from the first week (*p* = 0.009) to the 24-week follow-up (*p* = 0.005). Regarding the Barthel index, the between-group difference was significant only at the 24-week follow-up (*p* = 0.031), with the double ESWT group showing a greater mean change from baseline than the control group. Lastly, for strain elastography, a significant between-group difference was observed only in the first week (*p* = 0.009), indicating a softer tested muscle after double-dose ESWT treatment compared to the control ESWT treatment.

**Table 3:** Comparison of mean change from baseline in outcomes between double shockwave group and control shockwave group

Mean (SD)	Double SW group (*n* = 19)	Control SW group (*n* = 20)	*p*-value^†^
**Primary outcome: ΔMAS**			
1 week-baseline	-0.25 (0.86)	-0.33 (0.59)	0.719
4 weeks-baseline	-0.5 (0.82)	-0.33 (0.69)	0.693
12 weeks-baseline	-0.14 (0.66)	-0.06 (0.57)	0.716
24 weeks-baseline	-0.08 (0.64)	-0.14 (0.66)	0.804
**Secondary outcomes**			
**Δ R2-R1 Angle of MTS**			
1 week-baseline	1.88 (9.29)	3.33 (15.72)	0.999
4 weeks-baseline	-0.50 (9.13)	0.56 (14.34)	0.834
12 weeks-baseline	2.79 (8.83)	2.81 (14.72)	0.801
24 weeks-baseline	1.69 (7.84)	1.79 (12.34)	0.940
**ΔPROM**			
1 week-baseline	3.44 (8.11)	4.44 (12.94)	0.734
4 weeks-baseline	6.25 (9.92)	3.06 (13.52)	0.536
12 weeks-baseline	4.43 (7.49)	2.50 (15.28)	0.474
24 weeks-baseline	8.23 (11.43)	1.43 (13.07)	0.444
**ΔTimed Up and Go Test**			
1 week-baseline	-10.03 (14.53)	-1.44 (5.15)	0.009**
4 weeks-baseline	-7.22 (14.53)	-1.24 (6.53)	0.002**
12 weeks-baseline	-13.21 (11.75)	-5.14 (7.20)	0.015*
24 weeks-baseline	-15.23 (10.84)	-3.72 (7.54)	0.005**
**ΔBarthel index**			
1 week-baseline	8.44 (14.91)	3.33 (8.22)	0.436
4 weeks-baseline	13.75 (21.33)	3.34 (8.23)	0.070
12 weeks-baseline	8.93 (37.43)	4.06 (9.35)	0.196
24 weeks-baseline	17.69 (22.97)	4.29 (13.28)	0.031*
**ΔStrain elastography**			
1 week-baseline	-0.16 (0.37)	0.31 (0.58)	0.009**
4 weeks-baseline	-0.09 (0.43)	-0.10 (0.56)	0.730
12 weeks-baseline	0.06 (0.50)	-0.03 (0.67)	0.708
24 weeks-baseline	-0.02 (0.57)	-0.01 (0.62)	0.662

† Between-group comparison: Mann-Whitney U test was used for statistical analysis

The data was presented as mean (SD)

Abbreviation: SW, shockwave; MAS, Modified Ashworth Scale; MTS, Modified Tardieu Scale; PROM, passive range of motion; R1, angle of catch seen at quick speed; R2, Full range of motion at slow release of muscle; SD, standard deviation

*p < 0.05, **p < 0.01

Table 4 presents the GEE analysis results. For the primary outcome MAS, the analysis revealed no significant effects of the treatment group (*p* = 0.868), time (*p* = 0.722), or the interaction between group and time (*p* = 0.962). There were no significant effects of the treatment group on MTS (*p* = 0.163), PROM (*p* = 0.846), TUG Test (*p* = 0.688), Barthel index (*p* = 0.915), or strain elastography (*p* = 0.222). However, time significantly affected PROM (*p* = 0.014), TUG test (*p* < 0.001), and Barthel index (*p* < 0.001), suggesting improvements across all participants over the study period. Moreover, significant interactions between group and time were observed for the TUG test (*p* = 0.011), Barthel index (*p* = 0.036), and strain elastography (*p* = 0.008), indicating that the changes in these outcomes over time differed between the groups.

**Table 4:** Effect of shockwave on outcomes between groups and times: generalized estimation equation analysis

Outcome	*p*-value^†^		
	Group	Time	Group x Time
**Primary outcome: MAS**	0.868	0.722	0.962
**Secondary outcomes**			
**R2-R1 Angle of MTS**	0.163	0.157	0.494
**PROM**	0.846	0.014*	0.595
**Timed Up and Go Test**	0.688	< 0.001**	0.011*
**Barthel index**	0.915	< 0.001**	0.036*
**Strain elastography**	0.222	0.209	0.008**

† Generalized estimation equation analysis was used for between-time, between-group and group-time interaction

The data was presented as mean (SD)

Abbreviation: MAS, Modified Ashworth Scale; MTS, Modified Tardieu Scale; PROM, passive range of motion; R1, angle of catch seen at quick speed; R2, Full range of motion at slow release of muscle

*p < 0.05, **p < 0.01

## Discussion

This study investigates the dose-response effectiveness of focused ESWT on post-stroke ankle plantar flexor spasticity. Our within-group analysis revealed significant improvements in key clinical measures, such as the MAS, PROM, TUG test, and Barthel Index, in the double ESWT group throughout the follow-up period. The between-group analysis highlighted the superior performance of the double ESWT group, especially in reducing TUG Test times, improving the Barthel Index at the 24-week mark, and demonstrating an early reduction in muscle stiffness as shown by strain elastography. The GEE analysis further confirmed the superiority of the double-dose group in the TUG test, Barthel index, and strain elastography, suggesting a potential dose-response relationship. To our knowledge, this is the first prospective, randomized, double-blinded clinical trial to explore the optimal dosing and dose-response effectiveness of ESWT on post-stroke ankle plantar flexor spasticity.

Our study unveiled a significant improvement in MAS in the double ESWT group over the study period. The MAS did not significantly change after treatment in the control ESWT group (Table 2). In previous studies, the MAS has been a primary tool for assessing lower limb spasticity, with ESWT treatments leading to significant reductions in MAS scores. The MTS and Tardieu angles are utilized to evaluate spasticity changes [19–21, 28, 29, 37]. Notably, Wu et al. reported a 35% improvement in the Tardieu angle [29], while Aslan et al. observed a 29.8% improvement in the spasticity angle as measured by the Tardieu scale [28]. In our research, a significant decrease in spasticity was evident in the double-dose group, which aligned with the marked reduction in MAS reported in earlier studies [19–21, 28, 29, 37]. There was no significant change observed when employing the MTS in our study. This may be due to the lack of standardized protocols regarding test position, speed of stretch, number of stretch repetitions, and testing time [33, 38]. Variations in the stretch velocity and frequency, patient posture, and the nature of the reflexes measured by MAS and MTS could lead to these differences in results observed in our study [33]. 

The treatment and total number of sessions varied widely in previous research, including a single application, weekly sessions spanning three weeks, and twice-weekly sessions over two weeks [19, 21, 28, 29, 37]. The dose-dependent effectiveness observed in our study aimed to reinforce the association between higher energy flux densities in ESWT and more favorable therapeutic outcomes. It also highlighted the potential for optimizing ESWT parameters to improve the management of lower limb post-stroke flexor spasticity.

Our study demonstrates that ESWT has a beneficial impact on the TUG test outcomes. Within the double-dose ESWT group, there was a marked and statistically significant enhancement in TUG test performance. This group also outperformed the control group at all follow-up intervals, with the GEE analysis validating better results in those who received the double dose. The TUG test is a widely recognized measure for evaluating functional mobility and balance abilities [39]. Our consistent findings across different analyses highlight the efficacy of ESWT in improving mobility as measured by the TUG test and suggest the benefits of double-dose ESWT over 24 weeks. This sustained effect contrasted with the findings of Radinmehr et al., who reported a minimal, clinically insignificant 9.6% improvement after ESWT [21]. In comparison, our study revealed long-term improvements beyond an immediate response. Other research demonstrated significant increases in walking speed post-ESWT, as measured by the 10-meter walk test [40]. Conversely, Wu et al. found no improvement in the 10-meter walk test after an 8-week follow-up [29]. These differences could be attributed to stroke-related gait disturbances, often caused by spasticity and restricted ankle dorsiflexion [41]. 

Our research documented a significant increase in ankle dorsiflexion among those in the double-dose ESWT group, as measured by PROM, which likely contributed to their improved mobility. Our PROM of ankle dorsiflexion results were similar to other studies assessing ROM alterations post-ESWT [21, 42]. The observed decrease in intrinsic muscle stiffness and increased tissue extensibility due to ESWT might facilitate an improved PROM [29]. However, the divergent assessment tools and protocols across various studies lead to inconsistent results concerning the effect of ESWT on the gait pattern of people who have experienced stroke [19, 21, 29]. Therefore, future research should employ standardized methods and assessment instruments for a definitive evaluation of ESWT’s effects on gait performance among stroke survivors.

In our study, both groups showed notable improvements in the Barthel Index, highlighting ESWT’s potential to significantly enhance ADLs. This aligned with Taheri et al.’s findings of significant enhancements in the lower extremities functional scale [19]. Additionally, Aslan et al. discovered that while increased lower extremity function scores of the Modified Barthel Index were not initially evident, they became significant by the sixth week [28]. Furthermore, our between-group comparisons revealed pronounced improvements in the Barthel Index for the double-dose ESWT group throughout the follow-up period compared to the control ESWT group. The GEE analysis confirmed the superior performance of the double-dose ESWT group in the Barthel Index, indicating the benefits of ESWT on ADL functions over the whole follow-up period. Our findings suggested a dose-response relationship between ESWT and ADLs. Such outcomes could provide stroke survivors with improved functional independence and enhanced overall quality of life [28]. 

The muscle tested softer based on strain elastography after double-dose ESWT treatment compared to the control ESWT treatment in the early stage, but this difference was not observed in the long-term follow-up. This provides insight into ESWT’s physiological impact on muscle properties. Lee et al.‘s research employed ultrasound methods to track post-ESWT alterations, uncovering reductions in Achilles tendon length, muscle thickness, and pennation angle and an increase in muscle fascicle length. These changes were most significant at the four-week follow-up [37]. Similarly, Aslan et al. observed improvements in the muscle elasticity of the plantar flexor muscles in both the ESWT and control groups, but a marked improvement in clinical spasticity measures solely in the ESWT group [28]. Our previous review unveiled the commendable reliability of elastography to evaluate PSS, validated through its correlation with clinical measurements, and monitor the therapeutic response and efficacy of targeted muscles [43]. Our results underscored ESWT’s effect on the early decrease of muscle stiffness and muscle mechanics, leading to benefits in both clinical and elastographic evaluations. The consistency of our elastography findings with previous research highlights the importance of imaging techniques in assessing the impact of ESWT on muscle characteristics. This elucidates areas for further investigation, such as how ESWT may alter muscle structure and function, particularly post-stroke.

In our study, the primary outcome, MAS, showed significant improvement within the double-dose ESWT group but not between the groups, while secondary outcomes like the TUG test showed significant improvements both within and between groups. One possible explanation for this discrepancy is the effects of traditional rehabilitation, which both groups received, potentially masking the specific effects of ESWT and leading to non-significant differences in MAS between groups. The essential therapies to reduce PSS were traditional rehabilitations including range of motion exercises, muscle stretching and strengthening, stance and balance training, core stability exercises, gait training, functional training, the use of physical modalities, and orthoses [31]. Additionally, MAS, despite being widely used, has limitations due to its six-level ordinal scale, which might lack sensitivity to detect subtle changes in spasticity [44]. Furthermore, MAS cannot distinguish between dynamic shortening (exaggerated reflexes or clonus) and fixed shortening (stiffness or contracture) of a muscle [45]. In contrast, the TUG test provides a continuous measure, making it more sensitive and robust against masking effects from co-interventions. It detects smaller changes in functional mobility and provides a more reliable assessment of functional gains [46]. 

The strengths of this study include its double-blind design and comprehensive 24-week follow-up period. Previous studies typically have had a 12-week follow-up period. An extended follow-up allows for the assessment of ESWT’s long-term effects and sustained impacts. Our variety of assessment tools, including the MAS and Tardieu Scale for spasticity, the TUG Test for functional mobility, the Barthel Index for ADL, and elastography for examining muscle properties, further enriched our findings. The use of elastography added a novel dimension by evaluating the intrinsic and elastic structures of spastic muscles, offering a comprehensive understanding of ESWT.

This study had some limitations. First, extended treatment regimens may yield greater or more durable outcomes. Future studies should explore the effects of various numbers of ESWT sessions to identify optimal treatment strategies for sustained therapeutic results. Second, the study’s sample size, while adequate for preliminary exploration, could be increased in future research to improve applicability and provide further insight into ESWT’s effectiveness across diverse patient populations. Third, the variation in participants’ post-stroke phases was complex, suggesting the need to stratify participants based on their post-stroke timing for customized treatment protocols. Fourth, traditional rehabilitation may have masked the effects of ESWT, leading to non-significant differences in the primary outcome. Addressing these limitations is essential to advancing our comprehension of ESWT’s role in post-stroke rehabilitation and optimizing its clinical application.

The findings of our study indicate that double-dose ESWT provides better functional improvements compared to the standard single-dose ESWT, although both doses resulted in similar reductions in spasticity. Therefore, double-dose ESWT could be a potential choice for clinicians aiming to achieve better functional outcomes in patients with post-stroke ankle plantar flexor spasticity. Future research should explore varying frequencies, durations, and intensities of ESWT to determine the most effective parameters for treating post-stroke spasticity. Additionally, investigating the molecular mechanisms underlying the observed improvements could provide deeper insights into the treatment’s efficacy. Comparative analyses with other modalities such as botulinum toxin injections, physical therapy, or emerging technologies like repetitive transcranial magnetic stimulation could offer a comprehensive perspective on integrating ESWT into broader therapeutic practices. Developing an innovative artificial intelligence tool using ultrasound imaging for assessing spasticity, ROI identification, and 3D visualization holds promising potential to guide anti-spastic treatments and enable precise analysis of treatment effectiveness.

## Conclusions

Double-dose ESWT was more effective than a single-dose ESWT treatment in improving functional capabilities and elastography results. This study confirmed the dose-dependent effectiveness of ESWT in treating ankle-equinus spasticity among individuals who have survived a stroke.

## Data Availability

No datasets were generated or analysed during the current study.
